# Anomalous left coronary artery from the pulmonary artery: A case report of ALCAPA syndrome with bicuspid aortic valve

**DOI:** 10.1097/MD.0000000000042206

**Published:** 2025-04-18

**Authors:** Maaweya Jabareen, Wasef Alhroub, Mohammed Nassr, Hisham Nassar, Ahmad Emar, Zaid Alhusaini, Ahmad Hasasna

**Affiliations:** a Department of cardiology, College of Medicine, Hebron University, Hebron, Palestine; b Department of Cardiology, Al-Ahli Hospital, Hebron, Palestine.

**Keywords:** ALCAPA, bicuspid aortic valve, case report, congenital heart disease, medical management

## Abstract

**Rationale:**

Anomalous left coronary artery from the pulmonary artery (ALCAPA) syndrome, also known as Bland-White-Garland syndrome, is a rare congenital heart defect predominantly diagnosed in infants and associated with high early mortality rates without surgical intervention. While ALCAPA generally presents alone, it can sometimes occur alongside other congenital anomalies, such as a bicuspid aortic valve (BAV), a combination scarcely documented in the literature. This case report explores a rare presentation of ALCAPA syndrome in a 27-year-old adult with concurrent BAV.

**Patient concerns:**

The patient, a 27-year-old male, presented with progressive shortness of breath over several months, without additional symptoms such as chest pain or palpitations. His medical history was unremarkable, and he had no prior cardiovascular issues or significant family history of sudden cardiac death.

**Diagnoses:**

A physical examination revealed stable vital signs and an early diastolic murmur. Further investigations, including echocardiography and computed tomography coronary angiography, confirmed the diagnosis of ALCAPA syndrome with a BAV and mild to moderate aortic regurgitation. Cardiac catheterization demonstrated sufficient collateral circulation from the right coronary artery to the left coronary system, permitting adequate myocardial perfusion.

**Interventions:**

Due to the presence of adequate collateral circulation, the decision was made to manage the patient medically instead of surgically. The patient was started on a regimen of rivaroxaban, bisoprolol, and ramipril to manage his condition and reduce the risk of thrombus formation, in addition to providing myocardial protection.

**Outcomes:**

The patient responded positively to medical management, remaining asymptomatic with stable cardiovascular function at follow-up. After 2 years of follow-up, the patient continued to do well, with no reported symptoms.

**Lessons:**

This case emphasizes the importance of recognizing ALCAPA in adults, particularly in patients presenting with minimal symptoms despite significant congenital anomalies. In select cases with adequate collateral circulation, medical management may be a viable alternative to surgical intervention, underscoring the need for individualized treatment approaches based on the patient’s anatomical and functional profile.

## 1. Introduction

Bland-White-Garland syndrome or anomalous origin of the left coronary artery from the pulmonary artery (ALCAPA) is a rare congenital heart defect that typically presents with heart failure symptoms in infants. This condition occurs in about 1 in every 300,000 births. Without surgical treatment, nearly 90% of affected infants do not survive beyond their first year.^[1,2]^

Diagnosing ALCAPA in adults is exceptionally rare. Notably, even in the absence of symptoms, sudden cardiac death can occur, with around 90% of these patients experiencing sudden death by an average age of 35.^[3]^

Adults with ALCAPA may exhibit various symptoms, including chest pain, shortness of breath, palpitations, and irregular heartbeats, with some experiencing syncope or even sudden death. Interestingly, fewer than 15% of these individuals are asymptomatic.^[4]^

The typical approach to treatment is surgical reimplantation of the anomalous artery into the aorta. For patients diagnosed with ALCAPA, early detection and prompt surgical intervention are essential for a better prognosis.^[4,5]^

This case describes an adult patient with ALCAPA accompanied by a bicuspid aortic valve (BAV), who was managed with medical treatment. To our knowledge, this is only the second reported case in the literature of ALCAPA occurring in conjunction with a BAV.

## 2. Case presentation

A 27-year-old male presented to our private center with a primary complaint of shortness of breath that had gradually worsened over the past few months. He reported no associated symptoms, such as chest pain, palpitations, or lightheadedness. Over the last 5 years, the patient experienced intermittent fatigue but denied any previous cardiovascular issues or significant family history of sudden cardiac death.

On physical examination, the vital signs were stable: pulse 60 beats/min, blood pressure 125/77 mm Hg, and oxygen saturation at 95% on room air. There were no signs of cyanosis or peripheral edema. A precordial examination revealed an early diastolic murmur. The electrocardiogram was unremarkable, showing no signs of ischemia or arrhythmia.

**Figure 1.**
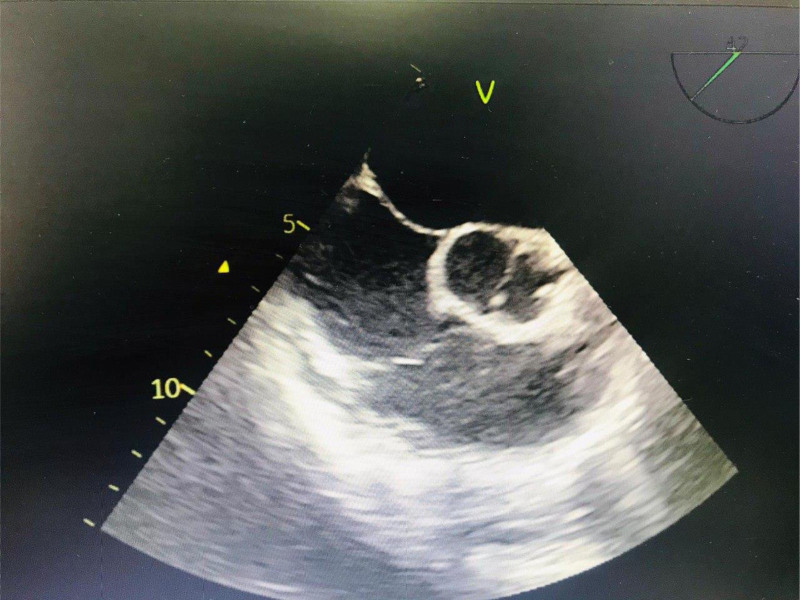
Transthoracic echocardiography showing bicuspid aortic valve.

To further evaluate his condition, transthoracic echocardiography was performed. These studies demonstrated a dilated left ventricle (left ventricular end-diastolic dimension of 6 cm) with borderline systolic function (ejection fraction of 50%) and a BAV with mild to moderate aortic regurgitation (Fig. 1). Given these findings, a computed tomography thoracic aorta study was conducted to rule out coarctation, which returned normal results.

**Figure 2.**
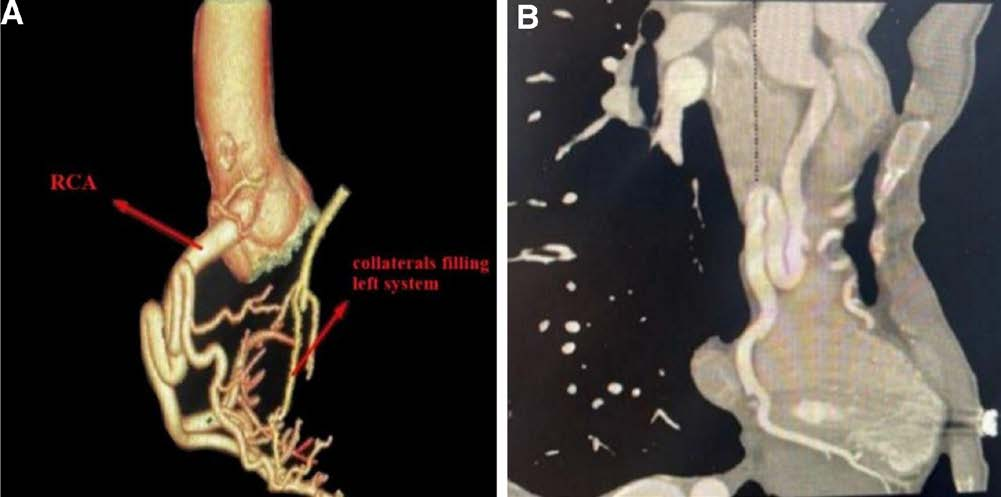
(A) Three-dimensional reconstruction of CT angiography images of the thoracic aorta. (B) Reconstruction for CT angiography of the aorta showing left main stem artery arising from pulmonary artery. CT = computed tomography.

Subsequently, computed tomography coronary angiography was performed to investigate. This imaging revealed a tortuous, dilated right coronary artery (RCA), which was supplying the left coronary system through multiple rich collateral vessels (Fig. 2A). Notably, the left coronary artery was found to be arising from the pulmonary trunk and was also dilated (Fig. 2B).

**Figure 3.**
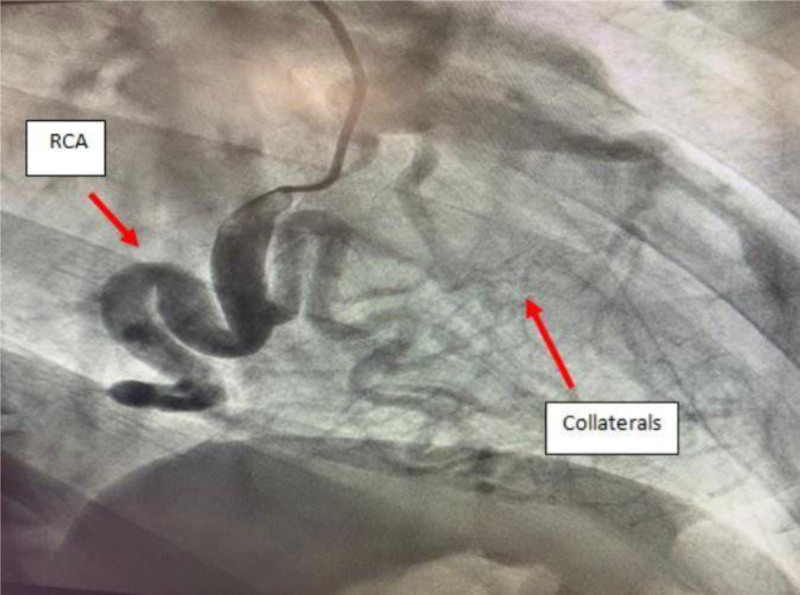
Coronary angiogram of the RCA, illustrating the RCA originating from the right coronary cusp, significantly dilated and supplying a network of rich collaterals to the left coronary system. RCA = right coronary artery.

**Figure 4.**
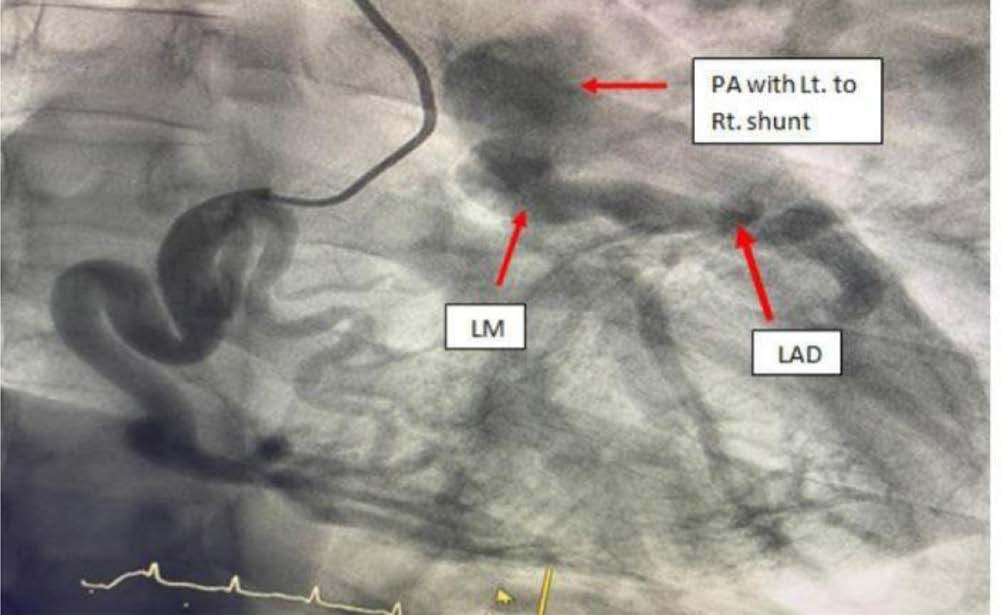
Angiogram of the left coronary system demonstrating substantial retrograde flow from the RCA, with the left main artery arising from the pulmonary artery instead of the left coronary cusp. RCA = right coronary artery.

Left and right cardiac catheterization were performed, revealing a markedly dilated RCA originating from the right coronary cusp and supplying an extensive collateral network to the left coronary system (Fig. 3). Additionally, the left coronary system (left main coronary artery, left anterior descending coronary artery, and left circumflex coronary artery) demonstrated substantial retrograde flow from the RCA, with the left main artery originating from the pulmonary artery rather than the left coronary cusp (Fig. 4). In other words, blood is directed from the RCA to the pulmonary artery through the left coronary system, and there is no flow from the pulmonary artery back to the left main coronary artery, indicating the absence of a left-to-right shunt.

The pressures recorded during catheterization were as follows: aortic pressure was 100/55 mm Hg (mean 75 mm Hg), left ventricular pressure was 100/15 mm Hg, right atrial pressure was 32/8 mm Hg, and pulmonary artery pressures were 32/13 mm Hg (mean 20 mm Hg). Oxygen saturation levels were measured at 96% in the aorta, 74% in the superior vena cava, and 78% in the pulmonary artery.

Ultimately, the patient was diagnosed with ALCAPA syndrome. Given the presence of adequate collateral circulation, surgical intervention was considered unnecessary. The patient was started on a medical management regimen that included rivaroxaban to mitigate the risk of thrombus formation due to the tortuous coronary anatomy, as well as bisoprolol and ramipril. After 2 years of follow-up, the patient continued to do well, with no reported symptoms.

## 3. Discussion

Anomalous origin of the left coronary artery from the pulmonary artery (ALCAPA) syndrome, commonly known as Bland-White-Garland syndrome, is a rare congenital heart defect first identified in 1933.^[6]^ While it often occurs in isolation, it can also be associated with other congenital heart conditions such as atrial septal defect, ventricular septal defect, patent ductus arteriosus, tetralogy of Fallot, aortopulmonary window, and coarctation of the aorta.^[7]^ To the best of our knowledge, the association of ALCAPA syndrome with a BAV has been documented in a single case in the literature in 2022.^[8]^

Embryologically, ALCAPA may arise from improper separation of the conotruncus into the aorta and pulmonary artery or from the persistence of the pulmonary buds alongside the involution of the aortic buds, which can form the coronary arteries. Additionally, a deficiency of vascular endothelial growth factor-C can prevent the cells of the capillary plexus surrounding the pulmonary artery and aorta from reaching or penetrating the normal coronary origins. As a result, the left coronary artery (LCA) and the left side of the heart receive blood from the pulmonary artery.^[7,9]^

The nature of ALCAPA results in the development of collateral circulation from the RCA to the anomalous left coronary artery, which arises from the pulmonary artery. This mechanism leads to left ventricular (LV) dysfunction. As a result, the onset of symptoms can vary based on the severity of the defect, the extent of collateral circulation, and the degree of LV dysfunction.^[10]^ In other words, patients like ours, who have sufficient collateral circulation from the RCA, may live into adulthood.^[11]^ Infants with ALCAPA commonly present with clinical signs of heart failure by approximately 3 months of age, as pulmonary arterial pressure diminishes. In the absence of appropriate treatment, the mortality rate is very high, with around 90% of affected infants not surviving beyond their first year of life.^[5]^

Our patient, a 27-year-old male, presented with worsening shortness of breath over several months but had no notable history of cardiovascular issues. He was diagnosed with ALCAPA and a BAV, which adds a unique dimension to our case. Despite his condition, he had sufficient collateral circulation, which contributed to his largely asymptomatic status.

Diagnosing ALCAPA in adults is rare. A 2011 study of 151 cases found the average age at diagnosis to be 41 years, with a female predominance of over 2:1. Sixty-six percent of patients experienced symptoms such as angina, dyspnea, palpitations, or fatigue. Seventeen percent presented with life-threatening events like arrhythmia or sudden death, while 14% were asymptomatic. Notably, 62% of those who experienced serious events had no prior symptoms. The average age for these life-threatening events was 33 years.^[12]^

Histological studies of the myocardium in ALCAPA patients, especially adults, are limited. Findings indicate variable degrees of fibrosis in biopsy samples from areas perfused by the anomalous artery. Many myocytes show viable characteristics, but a notable percentage exhibit reduced contractile material, reflecting delayed adaptive responses to chronic hypoperfusion.^[13]^ Electron microscopy reveals structural alterations in viable myocytes, suggesting adaptation to chronic ischemia, with abnormalities ranging from nearly normal to severely altered, depending on the examined site.^[13,14]^

The primary management for ALCAPA syndrome is surgical treatment, which focuses on restoring a normal 2-coronary-artery system. Early correction in neonates is vital for achieving significant improvements in ventricular function. However, in asymptomatic adults with moderate chronic ischemia and limited necrosis, survival without surgical intervention may also be feasible.^[15]^

The surgical management of ALCAPA involves 2 primary approaches: 1-coronary system repair and 2-coronary system repair.^[16]^ Single-coronary-system repair involves the ligation of the anomalous left coronary artery (LCA) at its pulmonary origin. In contrast, 2-coronary-system repairs include techniques such as coronary button transfer, the Takeuchi procedure, and coronary artery bypass grafting. The single-coronary-system approach is now largely avoided due to complications, including recanalization of ALCAPA, atherosclerosis, severe mitral regurgitation, and the risk of sudden death from silent ischemia. Ultimately, 2-coronary-system repairs provide improved outcomes and a lower risk of complications, making them the preferred choice in clinical practice.^[15,17]^

In our case, our decision to pursue medical treatment for this patient, which includes rivaroxaban, bisoprolol, and ramipril, is supported by findings suggesting favorable outcomes, including a lack of history of ischemic heart disease, the absence of severe left ventricular dysfunction, and the presence of sufficient collateral circulation. Additionally, the medical team has determined that if the patient exhibits symptoms related to the BAV that necessitate repair, both the aortic valve and the ALCAPA will be addressed in the same surgical procedure.

In general, congenital coronary anomalies are uncommon cardiac conditions characterized by abnormalities in the origin, course, or structure of the coronary arteries. For instance, in rare cases (0.03–0.50% incidence), the RCA may originate from the proximal ascending aorta instead of its usual anatomical location.^[18]^ Another variant is the anomalous origin of the RCA from the left sinus of Valsalva, which can cause cyclic compression and may lead to arrhythmias and cardiac ischemia. In the past, these anomalies often resulted in poor outcomes before they were diagnosed.^[19]^

## 4. Conclusion

This case report highlights a rare case of ALCAPA syndrome in an adult patient with a BAV, marking only the second documented case of this association in the literature. Despite his complex congenital heart defect, our patient remained largely asymptomatic due to adequate collateral circulation. This emphasizes the importance of recognizing ALCAPA in adults and suggests that, in certain cases, medical management can be a viable alternative to surgical intervention, particularly when significant ischemia is not present. Early detection and careful monitoring are crucial for optimizing outcomes in similar patients.

## Acknowledgments

We would like to thank the patient’s family for cooperating in this study.

## Author contributions

**Conceptualization:** Maaweya Jabareen, Wasef Alhroub.

**Data curation:** Maaweya Jabareen, Wasef Alhroub, Zaid Alhusaini.

**Resources:** Ahmad Hasasna.

**Software:** Ahmad Emar.

**Supervision:** Mohammed Nassr.

**Validation:** Mohammed Nassr, Hisham Nassar.

**Visualization:** Ahmad Emar.

**Writing – original draft:** Maaweya Jabareen.

**Writing – review & editing:** Maaweya Jabareen.

## References

[1] Talkhatova S Aripov M Mussayev A Alimbayev S Otarbayev Y Pya Y. ALCAPA in adult asymptomatic patient: a case report. Int J Surg Case Rep. 2023;109:108521. 37506526 10.1016/j.ijscr.2023.108521 PMC10413067

[2] Patel SG Frommelt MA Frommelt PC Kutty S Cramer JW. Echocardiographic diagnosis, surgical treatment, and outcomes of anomalous left coronary artery from the pulmonary artery. J Am Soc Echocardiogr. 2017;30:896–903. 28651802 10.1016/j.echo.2017.05.005

[3] Fierens C Budts W Denef B Van De Werf F. A 72 year old woman with ALCAPA. Heart. 2000;83:E2. 10618356 10.1136/heart.83.1.e2 PMC1729256

[4] Yi D Xia J Zhou X. Case report: a rare case of anomalous origin of the left coronary artery from the pulmonary artery accompanied with unilateral absence of pulmonary artery in an adult patient. Front Cardiovasc Med. 2023;10:1160893. 37153465 10.3389/fcvm.2023.1160893 PMC10157036

[5] Moodie DS Fyfe D Gill CC. Anomalous origin of the left coronary artery from the pulmonary artery (Bland-White-Garland syndrome) in adult patients: long-term follow-up after surgery. Am Heart J. 1983;106:381–8. 6869221 10.1016/0002-8703(83)90207-7

[6] Mazurak M Kusa J. HISTORY OF CARDIAC AND THORACIC SURGERY The radiologist’s tragedy, or Bland-White-Garland syndrome (BWGS). On the 80th anniversary of the first clinical description of ALCAPA (anomalous left coronary artery from the pulmonary artery). Pol J Cardio Thorac Surg. 2014;2:225–9. 10.5114/kitp.2014.43857 PMC4283871 26336427

[7] Blickenstaff EA Smith SD Cetta F Connolly HM Majdalany DS. Anomalous left coronary artery from the pulmonary artery: how to diagnose and treat. J Pers Med. 2023;13:1561. 38003878 10.3390/jpm13111561 PMC10672344

[8] Talebian Yazdi M Robbers-Visser D van der Bilt IAC. Anomalous coronary artery from the pulmonary artery diagnosed in adulthood: a case series on variations of coronary anatomy and the diagnostic value of cardiac magnetic resonance imaging. Puricelli F, Cecere A, Conte E, Anagnostopoulou A, Van den Eynde J, Djahit A, eds. Eur Heart J Case Rep. 2022;6:ytac345. 36045648 10.1093/ehjcr/ytac345 PMC9425847

[9] Boutsikou M Shore D Li W. Anomalous left coronary artery from the pulmonary artery (ALCAPA) diagnosed in adulthood: varied clinical presentation, therapeutic approach and outcome. Int J Cardiol. 2018;261:49–53. 29548537 10.1016/j.ijcard.2018.02.082

[10] Hu R Zhang W Yu X Zhu H Zhang H Liu J. Midterm surgical outcomes for ALCAPA repair in infants and children. Thorac Cardiovasc Surg. 2022;70:002–9. 10.1055/s-0041-1725978 33851407

[11] Al-Obaidi M Hecker F Walther T Holubec T. From asymptomatic adult patient to cardiopulmonary resuscitation – treatment of ALCAPA with total arterial myocardial revascularisation and mitral valve repair. J Cardiothorac Surg. 2024;19:369. 38918755 10.1186/s13019-024-02906-5 PMC11197221

[12] Yau JM Singh R Halpern EJ Fischman D. Anomalous origin of the left coronary artery from the pulmonary artery in adults: a comprehensive review of 151 adult cases and a new diagnosis in a 53‐year‐old woman. Clin Cardiol. 2011;34:204–10. 21462214 10.1002/clc.20848 PMC6652342

[13] Kubota H Endo H Ishii H. Adult ALCAPA: from histological picture to clinical features. J Cardiothorac Surg. 2020;15:14. 31931842 10.1186/s13019-020-1048-y PMC6958604

[14] Shivalkar B Borgers M Daenen W Gewillig M Flameng W. ALCAPA syndrome: an example of chronic myocardial hypoperfusion? J Am Coll Cardiol. 1994;23:772–8. 8113564 10.1016/0735-1097(94)90767-6

[15] Peña E Nguyen ET Merchant N Dennie C. ALCAPA syndrome: not Just a pediatric disease. Radiographics. 2009;29:553–65. 19325065 10.1148/rg.292085059

[16] Lange R Vogt M Hörer J. Long-term results of repair of anomalous origin of the left coronary artery from the pulmonary artery. Ann Thorac Surg. 2007;83:1463–71. 17383358 10.1016/j.athoracsur.2006.11.005

[17] Dodge-Khatami A Mavroudis C Backer CL. Anomalous origin of the left coronary artery from the pulmonary artery: collective review of surgical therapy. Ann Thorac Surg. 2002;74:946–55. 12238882 10.1016/s0003-4975(02)03633-0

[18] Ceresa F Micari A Mammana LF. Replacement of ascending aortic aneurysm with anomalous origin of the right coronary artery: multidisciplinary imaging for the diagnosis. J Cardiovasc Echogr. 2023;33:192–4. 38486693 10.4103/jcecho.jcecho_37_23 PMC10936709

[19] de Gregorio C Ceresa F Ferrazzo G Patanè F. Malignant right coronary artery origin from the left sinus of valsalva: complementary role for transesophageal echocardiography upon the cath‐lab diagnosis. J Clin Ultrasound. 2021;49:167–9. 32333791 10.1002/jcu.22845

